# Operational procedure sharing pathway in veno-occlusive disease: a Delphi consensus-based recommendations

**DOI:** 10.3389/fonc.2025.1498782

**Published:** 2025-03-12

**Authors:** Francesca Bonifazi, Federico Ravaioli, Anna Paola Iori, Giuseppe Milone, Attilio Olivieri, Arcangelo Prete, Domenico Russo, Stella Santarone, Simona Sica, Marco Zecca, Antonio Colecchia

**Affiliations:** ^1^ Department of Translational and Precision Medicine, Istituti di Ricovero e Cura a Carattere Scientifico (IRCCS) Azienda Ospedaliero-Universitaria di Bologna, Bologna, Italy; ^2^ Division of Allogeneic Transplantation, Department of Translational and Precision Medicine, “Sapienza” University of Rome, Rome, Italy; ^3^ Hematology and Bone Marrow (BMT) Unit, Azienda Ospedaliero Universitaria Policlinico “G.Rodolico-San Marco”, Catania, Italy; ^4^ Clinica di Ematologia, Università Politecnica delle Marche, Ancona, Italy; ^5^ Unit of Blood Diseases and Bone Marrow Transplantation, Cell Therapies and Hematology Research Program, Department of Clinical and Experimental Science, University of Brescia, Azienda Socio Sanitaria Territoriale (ASST) Spedali Civili di Brescia, Brescia, Italy; ^6^ Department of Hematology, Transfusion Medicine and Biotechnologies, Ospedale Civile, Pescara, Italy; ^7^ Dipartimento di Scienze di Laboratorio ed Ematologiche-Fondazione Policlinico Universitario Agostino Gemelli-IRCCS, Sezione di Ematologia, Rome, Italy; ^8^ Dipartimento di Scienze Radiologiche ed Ematologiche, Università Cattolica del Sacro Cuore, Rome, Italy; ^9^ Roma Department of Pediatric Hematology/Oncology, Fondazione IRCCS Policlinico San Matteo, Pavia, Modena, Italy; ^10^ Gastroenterology Unit, Dipartimento Chirurgico Medico, Odontoiatrico e di Scienze Morfologiche (CHIMOMO) Department University of Modena and Reggio Emilia, Modena, Italy

**Keywords:** veno-occlusive disease (VOD), Delphi, ETE, methodology, SOS

## Abstract

**Background:**

The hepatic Veno-Occlusive Disease (VOD), also known as Sinusoidal Obstruction Syndrome (SOS), is a serious complication that can occur after high-dose chemotherapy and hematopoietic stem cell transplantation (HSCT). In Italy, the approach to VOD varies due to differences in healthcare practices and diagnostic criteria among different regions.

**Aim and methods:**

To address this issue, a structured, multi-step Delphi consensus project was undertaken with the aim of standardizing the diagnostic and therapeutic pathways for VOD in Italian clinical practice. The project involved a methodologist, a scientific board of 10 experts, and an expert panel of 45 specialists from Italian hospital centers. This 12-month process included independent contributions, harmonization by a methodologist, and discussions through web meetings.

**Results:**

The survey identified 15 clinical topics divided into five key areas, including pre-HSCT patient evaluation, clinical-laboratory aspects for diagnosis and therapy, integration of clinical evaluations with EBMT criteria, monitoring with imaging techniques, and adherence to guidelines for managing defibrotide therapy. Key findings include the recommendation of weekly imaging even when VOD is not clinically suspected, the importance of early diagnosis and treatment with defibrotide, and the need for a standardized approach across different centers.

**Conclusion:**

The Delphi consensus revealed significant variability in the management of VOD across Italian centers and emphasized the necessity of a multidisciplinary approach involving hematologists, hepatologists, and radiologists. Establishing a national network for sharing best practices and utilizing advanced imaging technologies is essential for improving VOD diagnosis and treatment. The findings indicate the importance of implementing standardized protocols and continuous education to enhance patient outcomes in HSCT settings.

## Introduction

Sinusoidal obstruction syndrome (SOS), also referred as hepatic veno-occlusive disease (VOD; hereafter referred to as SOS/VOD), primarily manifests following high-dose chemotherapy, allogeneic hematopoietic stem cell transplantation (HSCT), and more rarely, due to ingestion of toxic alkaloid substances, high-dose non-transplant-associated chemotherapy, and liver transplantation (1, 2). It is a clinical syndrome characterized by rapid weight gain, fluid retention, ascites, and pain in the right hypochondrium associated with hepatomegaly and jaundice, typically occurring within thirty days after HSCT (though delayed occurrences have been noted) (3). Occasionally, cases of SOS/VOD may be identified in anicteric patients (especially in the pediatric population) (2, 4). The presence of the described symptoms defines the clinical diagnosis criteria for SOS/VOD.

Hepatic veno-occlusive disease (VOD) has been documented up to 60% of patients after HSCT, though reported incidence rates exhibit significant variability, from 5-10% up to 60%, across studies. This variability is influenced by factors such as transplant type, conditioning regimen, and diagnostic criteria as well (5–7).

Patients at higher risk for SOS/VOD include individuals with advanced disease stages, pre-existing liver diseases (resulting from viral infections, abdominal irradiation, or iron overload), exclusive parenteral nutrition during transplantation, exposure to hepatotoxic drugs, and those being conditioned with busulfan and cyclophosphamide preparing regimen (6, 7). SOS/VOD pathogenesis results from endothelial damage within hepatic sinusoids. The endothelial-damaged cells loose cohesion and facilitate the formation of gaps within the sinusoidal barrier. This allows red blood cells, leukocytes, and cellular debris to traverse through these gaps into the space of Disse beneath the endothelial cells, disrupting the endothelial lining. Subsequently, the venous lumen undergoes progressive narrowing (embolization of the centriolar vein), reducing sinusoidal venous outflow, ultimately resulting in post-sinusoidal portal hypertension (2). While SOS/VOD resolves gradually within few weeks in most patients, its most severe forms culminate into multi-organ failure (MOF) that is associated with a mortality rate exceeding 80% (5, 6, 8). Hence, despite its relatively low incidence, early detection of SOS/VOD is imperative, particularly with the advent of defibrotide, a novel drug demonstrating efficacy in its prevention and treatment (9–11).

Due to challenges in conducting invasive histological procedures for SOS/VOD diagnosis post-HSCT, clinicians have relied on surrogate clinical diagnostic criteria such as the modified Seattle and Baltimore criteria (12, 13). Despite recommendations (14), consensus documents (15, 16), and the definition of new diagnostic criteria (5, 17), the approach to SOS/VOD management in Italian clinical practice remains only partially standardized.

Factors contributing to this heterogeneity include the adoption of different diagnostic criteria, ranging from older ones [Baltimore and modified Seattle (12, 13)] to more recent ones [EBMT (5, 7, 15, 18)], in which ultrasonography with color Doppler supports clinical evaluation, enabling very early (pre-clinical) diagnosis, especially in late-onset forms. Moreover, similar to other HSCT-related complications, evidence regarding prognosis and treatment strategies is scarce and often weak, primarily derived from anecdotal reports, retrospective analyses, and non-randomized prospective studies (8). Finally, the regionalization of the Italian Healthcare System, with different criteria regulating access to medications, hampers the standardization of therapy protocols and patient management. In this context, the “Operational Procedure Sharing Pathway in SOS/VOD” project aims to explore and describe the clinical-diagnostic-therapeutic management of hepatic veno-occlusive disease in clinical practice (adult and pediatric patients) in Italian hospital facilities. Specifically, this project seeks to ascertain whether there is a common approach to identifying and managing post-transplant SOS/VOD patients and assessing the risk of progression.

The project involved several key figures:

- Methodologist: It is essential to ensure full compliance and adherence to the selected scientific method and harmonization of outputs produced by the Scientific Board’s discussion.
- Scientific Board: Tasked with identifying topics to focus on and constructing a survey to query the Expert Panel, selected from Italian Hospital Centers involved in HSCT patient management and handling associated complications.
- Expert Panel: Comprised of representatives from Italian Hospital Centers performing HSCT. The Panel, responding to multiple-choice questions proposed by the Scientific Board, represents the Italian reality of various Centers regarding SOS/VOD management.

## Methods

The Delphi-based process, with its Estimate-Talk-Estimate framework, allows structured communication and collaboration among experts. This leads to the refinement and synthesis of diverse opinions into collective judgments or recommendations on the topic at hand (19, 20). The method selected for this project is the Estimate-Talk-Estimate approach in the mini-Delphi format.

The comprehensive process spanned 12 months and was structured around three key steps (step 1, step 2 and step 3), all adhering to a unified approach. Firstly, individual members of the scientific board were engaged in independent and anonymous work to contribute their insights and perspectives. Secondly, the methodologist-facilitator took charge of harmonizing and synthesizing the diverse inputs received, ensuring coherence and clarity in the collective output. Finally, a web meeting provided a platform for collaborative sharing, enabling robust discussions and the exchange of ideas among the participants. Crucially, these phases were designed to be iterative, allowing multiple repetitions until a consensus was achieved.

### Step 1 identification of topics (clinical scenarios worthy of investigation) for discussion

The scientific board panel comprised 10 experts in the field (hematologists with competences on allogeneic HSCT). Each board member independently proposed a list of clinical scenarios/topics worthy of investigation. These scenarios encompass aspects related to the accurate identification and management of post-transplant patients eligible for SOS/VOD treatment, as well as the assessment of the risk of progression, which are unclear, worthy of further investigation, or present conflicting scientific evidence. Each Board member sent their points of interest to the methodologist, who undertook the harmonization of the received contributions to finally generate a definitive list of clinical scenarios worthy of investigation with the scientific board members.

### Step 2 construction of the survey to be submitted to the expert panel

Each board member autonomously and independently formulated the defined clinical scenarios as multiple-choice questions. The methodologist then harmonized the formulated multiple-choice questions to finally generate a definitive list of 15 clinical topics (referred to as “items” in the results narrative) worthy of interest in the form of questions/answers to present to the expert panel members.

### Step 3 the survey is presented to the expert panel members

45 specialists in the field were identified at the Italian national level, representing thematic areas and different realities within the territory. The online survey, which began on September 28, 2021, and ended on January 15, 2022, saw the participation of 34 expert panel members distributed throughout the national territory. Each participant in the expert panel had to independently choose the option most consistent with their daily clinical practice for each clinical scenario under investigation or provide their own response if they did not find the most adherent option to their clinical practice among the selected options. The methodologist collected and graphically elaborated on all the answers to provide the scientific board with the assessment of results across the entire national territory. During a subsequent meeting, the board proposed a comment and a critical evaluation of the 15 clinical topics.

## Results

The results related to 5 areas deserving of greater attention are reported first, emerging as priorities, based on the results of the present survey and on the most updated literature. The 5 areas and the different items for each single area by a discussion deepening requested by the members of the Scientific Board were reported in **Table 1**. For each item, the Expert panel was asked to choose the option most relevant to their clinical practice.

**Table 1:** Areas and items included in the Delphi consensus.

AREA 1 - Evaluation of the patient prior HSCT
Items 1 - How should clinicians manage patients at increased risk of VOD who have been exposed to drugs known to potentially induce VOD?
Items 2 - How should clinicians manage patients with a history of liver disease (hepatitis, steatosis, hemochromatosis, cirrhosis with or without portal hypertension)?

AREA 2 - Identification of clinical-laboratory aspects for defining a correct diagnosis and subsequent appropriate therapy.
Items 3 - In the case of worsening hyperbilirubinemia (bilirubin >2 mg/dl) in the absence of other clinical criteria, what should be the next steps in patient management?
Items 4 - In the case of rapidly evolving hypertransaminasemia combined with worsening hyperbilirubinemia (bilirubin >2 mg/dl), what are the recommended next steps in patient management?"
Items 5 - In the case of platelet refractoriness, what should be the clinical approach to management and treatment?
Items 6 - In the case of water retention (increase in body weight >5%), when do you consider it resistant to therapy after performing?
Items 7 - In the case of renal function disorders and hypernatremia states (accompanied by weight gain), what is the recommended clinical management approach?

AREA 3 - Integration between clinical evaluations and EBMT criteria.
Items 8 - In a clinical picture suggestive of VOD but lacking complete EBMT criteria 2023, what should be the diagnostic and therapeutic approach?
Items 9 - In the presence of an ultrasound picture indicative of classic VOD with or without clinical criteria, what should be the clinical approach to diagnosis and treatment?
Items 10 - In the presence of atypical VOD (without jaundice or late onset), what should be the clinical approach to diagnosis and treatment?

AREA 4 - Role of monitoring with imaging techniques (Doppler ultrasound, abdominal ultrasound, liver elastography) in transplant patients and those undergoing defibrotide therapy.
Items 11 - What is the recommended protocol for monitoring transplant patients using imaging techniques such as echo Doppler, abdominal ultrasound, and liver elastography (FibroScan)?
Items 12 - What is the recommended protocol for monitoring patients receiving defibrotide treatment using imaging techniques such as echo Doppler, abdominal ultrasound, and liver elastography (FibroScan)?

AREA 5 - Compliance with product sheet guidelines for managing Defibrotide
Items 13 - In patients exhibiting an early response to defibrotide treatment within 7 days, what is the most appropriate clinical management strategy?
Items 14 - In the event of persistent SOS/VOD after the standard 21 days of treatment with defibrotide, what is the appropriate clinical management strategy?
Items 15 - What concomitant therapies are considered or recommended when administering defibrotide?

### Area 1 - evaluation of the patient prior HSCT

Item 1 - How should clinicians manage patients at increased risk of VOD who have been exposed to drugs known to potentially induce VOD?

Item 2 - How should clinicians manage patients with a history of liver disease (hepatitis, steatosis, hemochromatosis, cirrhosis with or without portal hypertension)?

#### Panelists’ view and behavior in VOD clinical practice

This first area explored risk factors before HSCT (Item 1) and in patients with a history of liver disease (Item 2). In addition, the panelists’ behavior in case of exposure to potentially high-risk VOD drugs was also investigated. In this case, 44% of the participants stated that they would start the transplantation without prophylaxis but with close follow-up; 35% would change the conditioning regimen according to the risk of VOD; 3% would delay the transplantation after a suitable period since the last drug administration. Importantly, 18% of participants would adopt different behaviors, such as prophylaxis with defibrotide, reducing the conditioning regimen or using heparin. Regarding cases of previous liver disease, 44% of the panel members would start transplantation with close follow-up, 32% would start UDCA first, 6% would perform a liver biopsy, and 6% would not perform a transplant procedure.

#### Comments from the scientific steering committee

The panel emphasized the importance of risk factor assessment for VOD in HSCT patients. Guidelines recommend a thorough evaluation and documentation of these factors in the clinical chart, with special attention to prior exposure to drugs like gentuzumab or inotuzumab ozogamycin. The use of oral busulfan, though largely discontinued, alongside myeloablative total body irradiation (TBI) or multiple alkylating agents, necessitates close monitoring. There is no evidence linking checkpoint inhibitors to an increased risk of SOS/VOD. Key risk factors also include pre-existing liver conditions, such as HBV or HCV viremia, or elevated transaminase levels.

Despite the strength of recommendations on risk factors, the available literature is mainly based on retrospective studies. Prophylactic treatment with antiviral agents is recommended in patients with HBsAg+ or antiHBc. Prior HBV or HCV in the era of antiviral agents should be considered eligible for HCT without restriction if there is no evidence of severe liver dysfunction (i.e., cirrhosis) according to Child-Pugh criteria. The panel confirmed the need for an interval of at least 30 days in case of prior exposure to inotuzumab. Prophylaxis with UDCA 12mg-15mg/kg/die from the conditioning regimen and up to day+100 after HCT, tested in 3 randomized trials and a systematic review (21–23), is widely adopted in all centers in Italy (8). On the other side, however, the guidelines from BCSH/BSBMT attribute a low level of strength (14). Defibrotide prophylaxis is not indicated in adult patients: results from the randomized trial Harmony, recently published, fail to achieve its primary endpoints (VOD/SOS free survival at day 30 after HSCT). For this reason, EMA recently raised an issue against the use of defibrotide in prophylaxis (11). However the prior exposure to drugs potentially associated with an increased risk of VOD is not yet considered as a contraindication to allo HSCT. The panel considered instead strict follow-up of these patients, including prophylaxis with UDCA (24).

Modifications to the conditioning regimen, such as using treosulfan instead of busulfan, despite being considered in the real life setting, require caution, since no evidences can be currently provided. Indeed, modifying the conditioning regimen is not felt to be correct without solid evidence (8). In case of prior liver disease, the Panel agrees on a multidisciplinary evaluation with active collaboration with a hepatology/gastroenterologist, including the discussion about liver biopsy, if appropriate Cirrhosis was felt to be either a contraindication or a very high-risk factor prior to allogeneic HSCT. Prophylaxis with defibrotide was not considered feasible in this setting, although but in the pediatric one no consensus was finally reached.

### Area 2 –identification of clinical-laboratory aspects for defining a correct diagnosis and subsequent appropriate therapy

Item 3 - In the case of worsening hyperbilirubinemia (bilirubin >2 mg/dl) in the absence of other clinical criteria, what should be the next steps in patient management?

#### Panelists’ view and behavior in VOD clinical practice

Approximately 80% of panel members would evaluate radiological signs of VOD by ultrasound and elastography; another 18% would evaluate by ultrasonography and elastography for radiological signs of VOD but would start defibrotide treatment independently of the results, and the last 3% would reassess the medical history to evaluate VOD risk factors.

#### Comments from the scientific steering committee

The questions for Area 2 are focused on identifying clinical-laboratory aspects such as isolated hyperbilirubinemia and platelet refractoriness as drivers for defining VOD diagnosis and appropriate therapy. Although hyperbilirubinemia and platelet refractoriness are two undefined VOD criteria so far, hyperbilirubinemia is known to be the major discriminating factor between the Baltimore and Seattle criteria for VOD (12, 25, 26).

For these reasons, the EBMT Group proposed 2016 two different diagnostic criteria and a scale of VOD severity (5, 17). In adult patients, a distinction was made between traditional onset (within 21 days post-transplant day) and a late onset VOD (5). In the pediatric population, increasing bilirubin levels have not been considered mandatory for diagnosis of VOD (17).

Anyway, although the increasing expertise developed in these years, the early diagnosis of SOS/VOD remains challenging in some patients who still need to fulfil all SOS/VOD criteria despite having severe disease. This situation can lead to delayed initiation of treatment, which may have life-threatening consequences. In 2023, the EBMT criteria were updated to include a new category of “probable” SOS/VOD, defined by the presence of at least two out of five criteria: hyperbilirubinemia, painful hepatomegaly, weight gain greater than 5%, ascites, or ultrasound/elastography findings suggestive of SOS/VOD (Table below) (27). This update mitigate delays in diagnosis that could lead to life-threatening complications if treatment is not initiated promptly.

Importantly, these criteria overlap with the revised EBMT criteria for late-onset SOS/VOD. Therefore, the distinction probable/clinical/proven will also be applied, and the only difference for diagnosis between classical and late-onset SOS/VOD will be the time of onset (up to day 21 or after day 21).

Item 4 - In the case of rapidly evolving hypertransaminasemia combined with worsening hyperbilirubinemia (bilirubin >2 mg/dl), what are the recommended next steps in patient management?”

#### Panelists’ view and behavior in VOD clinical practice

The purpose of this item was to investigate the behavior of the panel in case of rapidly increasing hypertransaminasemia and bilirubinemia >2 mg/dl; almost all the panel experts would carry out further imaging and laboratory investigations to evaluate other signs of VOD and possibly exclude other causes.

#### Comments from the scientific steering committee

The rapid increase of transaminases does not represent a diagnostic criterion of VOD both in adults and in children; however, the EBMT consensus for the new diagnostic criteria of VOD in adults and children (17, 27) included both the rate of increase of serum bilirubin and hypertransaminasemia variations among the indexes of VOD severity. As the levels of transaminases appear to be a relevant parameter to evaluate liver dysfunction, cut-off points have also been defined to reflect this correlation. EBMT consensus 2023 (27) identified different ranges of hyperbilirubinemia and hypertransaminasemia: for the severe VOD, bilirubin can reach values from 5 to 8 mg/dl or doubling within 48 h, while transaminases could be included between 5 and 8 times the normal values. Moreover, the risk of developing a severe VOD in a patient whose serum bilirubin level increases from 3 to 6 mg/dL within 48 h is higher than that of another one who reaches this level over a more extended period (25, 28); similarly to the serum bilirubin increase, the liver failure (generally associated with worsening hypertransaminasemia) reflects the severity of VOD (29). However, attention must be paid to other possible causes of the rapid increase of serum transaminases and bilirubin levels (29).

The VOD severity was primarily assessed based, retrospectively, on multiorgan failure (MOF) and on survival outcomes (30, 31). The EBMT consensus has proposed new criteria for grading VOD severity in adults (and children) based on clinical and pathologic factors (17, 27). Signs and symptoms of potential VOD include rapid weight gain, oedema, ascites, painful hepatomegaly, hyperbilirubinemia and other indicators, including thrombocytopenia, renal insufficiency or encephalopathy (6). Following diagnosis of VOD, gauging its potential severity is essential for initiating appropriate treatment, and the kinetics of symptoms onset is also necessary for evaluating the severity of VOD. A patient whose symptoms emerge within days is much more likely to develop a severe VOD than one whose symptoms emerge over one or more weeks (32). Therefore, a criterion evaluating the time from the date when the first signs of VOD began to appear (retrospectively determined) and the date when the diagnosis of VOD should be based not only on bilirubin variations but also on other liver functional test deterioration, particularly a quickly worsening hypertransaminasemia, both in adults and in children.

Item 5 - In the case of platelet refractoriness, what should be the clinical approach to management and treatment?

#### Panelists’ view and behavior in VOD clinical practice

65% of the panelists would assess the presence of antibodies anti-HLA or anti-platelets and other VOD signs or symptoms; 26% of them would perform abdominal ultrasound other than platelet transfusions over 24 hours.

#### Comments from the scientific steering committee

From a definition point of view, an absolute platelet increment of less than 10x10^9^ per L 24 h after apheresis unit in an adult should be considered platelet refractoriness (PR) (33). In adult patients, this parameter alone is not yet deemed diagnostic for VOD but a red flag should be put on it. In fact, most panelists would do additional exams (imaging, Ac Anti HLA or platelets) and search for other signs of VOD. Indeed, PR is now considered diagnostic criteria only in pediatric patients, and it has been included in EMBT pediatric guidelines (17). Regarding PR as a diagnostic criterion of VOD in adults, studies are few and inconsistent; in fact, in the past, some small studies have observed an increase in the need for transfusions before the onset of signs of VOD and more evident in the severe form (34) and, a significant difference in transfusion requirements between VOD and control patients (35). However, Jones et al. found no difference between patients with and without VOD in the frequency of platelet refractoriness (12). To date, PR is not considered a predictor of VOD in the adult population,

Item 6 - In the case of water retention (increase in body weight >5%), when do you define it resistant to diuretic therapy?

#### Panelists’ view and behavior in VOD clinical practice

The totality of panelists believes that water retention could be considered resistant after treatment with albumin infusion and a high dose of furosemide. In fact, 94% of them replied that in the case of resistant water retention, the patients should undergo closing monitoring, high diuretic dose administration, fluid restriction intake and albumin infusion. 3% of the panelists added evacuative paracentesis to the previous advice, while another 3% would advise only closing monitoring and high diuretic dose.

#### Comments from the scientific steering committee

In allogeneic HSCT patients, the endothelial liver sinusoid damage, following the conditioning regimen and several additional triggers, determines a structural alteration of the liver sinusoids, which swell and go to apoptosis. This process determines loss of endothelial cell cohesion, triggering of inflammatory phenomena, recruitment of leukocytes, red blood cells, inflammatory cells and pro-coagulant factors in the space of Disse, causing post-sinusoidal hypertension (8). Splanchnic vasodilatation, a direct result of portal hypertension, leads to a decrease in effective arterial blood volume (EABV), which then activates homeostatic systems such as the renin-angiotensin system and sympathetic nervous system to promote sodium and water retention and vasoconstriction. The development of ascites is due to increased hydrostatic pressure and capillary permeability in splanchnic capillaries (36). For these reasons, fluid retention with renal failure may gradually develop in patients with VOD, and from a pathophysiologic point of view, it resembles hepatorenal syndrome in many ways. Early symptoms include peripheral oedema, ascites that gradually won’t respond to just diuretic treatment, sodium retention, weight gain, liver failure and hyperbilirubinemia. Endothelial damage progresses to MOD (Multi Organ Disease) when severe. Acute kidney insufficiency (AKI) may start slowly and progress; it commonly appears 10 to 16 days after HCT and may be brought on by conditions including hypotension, infection, or exposure to nephrotoxic agents. Upon diagnosis of VOD, prompt measures should be taken aimed at limiting the evolution of VOD into Multi Organ Disease, such as reduction of liquid intake, close monitoring of body weight, maintaining sodium and water balance, preserving renal blood flow, and managing peripheral oedema and ascites, with the judicious use of diuretics. Therapeutic paracentesis could be needed in more advanced phases other than diuretics with albumin supplementation. In patients with large fluid intake requirements, fluid management can be particularly challenging, and renal replacement therapy may be necessary (1, 29).

Item 7 - In the case of renal function disorders and hypernatremia states (accompanied by weight gain), what is the recommended clinical management approach?

#### Panelists’ view and behavior in VOD clinical practice

The response of the panelists has been quite heterogeneous; in fact, 26% of them would perform renal ultrasound and adjustment of the drug according to creatinine clearance, 21% would withdraw nephrotoxic drugs, 18% would increase fluid hydration together to high diuretic dose administration, 9% would perform renal ultrasound and adjustment plasmatic sodium levels, last 28% would do other strategies such as ultrasound, fluid restriction, plasmatic sodium correction or withdraw of potentially nephrotoxic drug.

#### Comments from the scientific steering committee

As discussed in item 6, renal damage is a direct consequence of portal hypertension, and its degree could also be influenced by arterial hypotension, infections, nephrotoxic agents, inappropriate diuretics use, pre-existing chronic kidney disease, diabetes, ageing, etc. Even with heterogeneous replies, our panelists have highlighted the role of fluid and kidney dysfunction management and electrolyte balance as the main interventional issues.

In particular, since VOD renal damage could also be drug-induced, in case of impaired renal function and contextual hypernatremia with weight gain, the better approach is to make a dosage adjustment of drugs, based on creatinine clearance (37). However, this therapeutic response is often more complex than expected; in most cases, in fact, it involves “life-saving” drugs in the patient’s post-transplant pathway, such as immunosuppressive drugs (cyclosporine/tacrolimus, methotrexate) and nephrotoxic antibiotics and antivirals (37). For these reasons, the correction of hypernatremia is quite complex because the balance of fluid and electrolytes is often in the context of renal failure, and it should be managed with the nephrologist (38). In clinical practice, therefore, VOD management needs:

- careful clinical (weight-vital parameters-input/output balance) and laboratory monitoring (blood count and hepato-renal function, electrolyte, daily) (8, 39);
- instrumental monitoring by liver ultrasound + elastosonography + renal ultrasound; x-ray or chest CT and echocardiogram in case of coexisting cardiovascular system impairment (39);
- Dosage adjustment of nephrotoxic drugs, correction of hypernatremia, and support of the Nephrologist specialist, especially in Severe and Very Severe VOD forms (38).

### Area 3 - integration between clinical evaluations and EBMT criteria

Item 8 - In a clinical picture suggestive of VOD but lacking complete EBMT criteria 2023, what should be the diagnostic and therapeutic approach?

#### Panelists’ view and behavior in VOD clinical practice

The 59% of panelists would start defibrotide treatment anyway, while about 30% of them would wait for the evolution of the clinical course until concordance between clinical and diagnostic criteria.

#### Comments from the scientific steering committee

EBMT criteria were developed by the European Group of Bone Marrow Transplantation and Cellular Therapy (EBMT) several years ago, defining peculiar features (and criteria) for adults (updated by the new revised EBMT criteria 2023 for adults) and pediatric settings (17). The backbone of the new diagnostic criteria relies on the Baltimore and Seattle criteria, but, in particular, for the children, it includes more relaxed criteria (i.e. no time limit for the onset). Furthermore in the adult updated EBMT criteria, ultrasonography and elastometry can identify a probable VOD.

The panelists, in the absence of a specific imaging monitoring protocol, highlighted the crucial role of the nurses who are required to evaluate specific parameters leading the transplant team to capture early symptoms and signs (40), in order to make an earlier diagnosis and improve the outcome (41).

For this reason, 59% of the panel answered to start the treatment anyway, even in case of not completeness of EBMT criteria but with a high-grade suspicion; this approach can be justified only in high-risk patients with several risk factors and not all patients. Such an observation explains why 29% of the panelists answered to wait until specific examinations are available and diagnostic criteria are fulfilled. Some panelists underlined the need to exclude differential diagnoses, even with invasive approaches such as HVPG. This point deserves attention because portal hypertension is a crucial point for VOD development. The ELASTOVOD study (*ClinicalTrials.gov NCT03426358)* will help in these cases since elastometry could fully abrogate invasive approaches aimed to measure portal hypertension.

Item 9 - In the presence of an ultrasound picture indicative of classic VOD with or without clinical criteria, what should be the clinical approach to diagnosis and treatment?

#### Panelists’ view and behavior in VOD clinical practice

Item 9 focused on specific therapy start in those patients with an ultrasonographic (US) diagnosis of VOD in the absence of confirmed clinical diagnostic criteria fulfilment. 47% of the panelists would start treatment with defibrotide; 29% would start treatment despite no significant bilirubin increase (were less than 2 mg/dl); 15% would start treatment but be prepared to discontinue it if the bilirubin value decreased (<2 mg/dl). Nine per cent would adopt several center-specific strategies, (discontinuation of defibrotide prematurely if the clinical picture resolves, as well as HVPG measurement and wait and see approach).

#### Comments from the scientific steering committee

The point is controversial. VOD is not a US diagnosis but a clinical diagnosis, even though the US is one of the criteria for probable VOD diagnosis, according to EBMT 2023. Anyway, an increase of bilirubin is mandatory for the “clinical diagnosis of VOD” in the presence of the other two criteria as hepatomegaly, weight increase and ascites. The duration of therapy is 21 days, independent of the response. In case of non-fulfilment of diagnostic clinical criteria with US diagnostic for VOD, the diagnosis remains at the physician’s discretion and the ability to examine to exclude the potential differential diagnosis. Some panelists answered to discontinue therapy if bilirubin is normal, even before day 21. However, no evidence of the appropriateness of such an approach can be found in the literature; therefore, it is not recommended, and particular caution should be put on this.

Item 10 - In the presence of atypical VOD (without jaundice or late onset), what should the clinical approach be for diagnosis and treatment?

#### Panelists’ view and behavior in VOD clinical practice

Item 10 explores the behavior of panelists in the case of atypical VOD; approximately 44% of panel members will have further evaluation by imaging (abdominal ultrasound, Doppler ultrasound, and elastography) and laboratory tests (including assessment for the presence of platelet refractoriness) and body weight monitoring to confirm the diagnosis of VOD sooner to start with a specific treatment. Another 26% would evaluate imaging and laboratory tests before beginning any treatment, and 15% of panel members would start defibrotide along with ultrasound monitoring.

#### Comments from the scientific steering committee

Some diseases, in particular in the pediatric setting, such as thalassemia, sickle cell disease, hemophagocytic lymphohistiocytosis or osteopetrosis, may present pre-existing hepatomegaly hyperbilirubinemia and ascites before transplantation. Since hyperbilirubinemia in children is frequently either absent or found only in advanced-stage severe VOD (34, 42, 43) this diagnostic criterion is difficult to use. For these reasons, pediatric EBMT consensus recognizes anicteric VOD as a frequent entity, with hyperbilirubinemia as a non-mandatory criterion. Instead of a predefined level of hyperbilirubinemia in children, the new EBMT criteria require the bilirubin level to rise from an individual baseline on 3 consecutive days or ≥2 mg/dl within 72 h after having excluded competing causes (17).

Most reports on VOD incidence and outcome before the new consensus publication based on Seattle and Baltimore criteria. The main difference between the two classifications is about hyperbilirubinemia, which was mandatory in Baltimore but not in the modified Seattle criteria. Hyperbilirubinemia and jaundice are rarely absent in adults with classical VOD, typically occurring within the first 21 days after HHCT. Still, even though hyperbilirubinemia can be delayed (occurring lately after liver pain and fluid retention), this sign can be absent in VOD that develops later (44). In the past most adult HSCT centers preferred Baltimore criteria, including in the setting of prospective clinical trials (6); this may represent a problem in patients who develop late onset VOD in the absence of hyperbilirubinemia, with only weight gain and ascites (45). In fact Myers et al. (42) retrospectively reviewed 794 HHCT patients, identifying 17 (2.1%) who developed VOD; of these, 5 (29%) did not have elevated bilirubin at VOD diagnosis. With the new EBMT criteria 2023 (27), which are dedicated to the adult setting, the classical and late-onset VOD differentiate only by the time of onset; hyperbilirubinemia is one of the parameters for the probable VOD and remains mandatory for diagnosing clinical VOD. However in difficult cases trans jugular liver biopsy considered gold standard (but not mandatory) for VOD diagnosis (46–48) and included in the new EBMT criteria, could be performed, but with caution due to its potentially life-threatening side effects.

### Area 4 - role of monitoring with imaging techniques (doppler ultrasound, abdominal ultrasound, liver elastography) in transplant patients and those undergoing defibrotide therapy

Item 11 - What is the recommended protocol for monitoring transplant patients using imaging techniques such as echo Doppler, abdominal ultrasound, and liver elastography?

The purpose of this fourth topic of the questionnaire was to investigate how imaging techniques (abdominal ultrasound, doppler ultrasound and liver elastography) should be used to monitor all patients undergoing HSCT as well as in those under defibrotide treatment of VOD development. Indeed, item 11 explores how imaging techniques should be planned before and after HSCT as per standard practice.

#### Panelists’ view and behavior in VOD clinical practice

56% of experts participating in the survey stated that they performed imaging techniques once a week but only if clinical suspicion of VOD was present, 29% once a week even in the absence of VOD suspicion, 3% weekly but only in patients in treatment with defibrotide and 12% at other different times: i) never, ii) once a week but only elastography, iii) both ultrasound and elastography before HSCT and after HSCT once a week in VOD suspicion.

#### Comments from the scientific steering committee

The Scientific Committee believed that the most consistent response would be to perform the imaging technique weekly, even without clinical suspicion.

The diagnosis of VOD has historically always been a clinical diagnosis, even though the latest position papers and guidelines (5, 14, 17) have emphasized the key role of imaging in helping physicians perform an early VOD diagnosis. Indeed, the European Society for Blood and Marrow Transplantation proposed new diagnostic criteria for adults in 2016, revised and updated in 2023 (27), and for pediatric patients in 2018 (17). These EBMT criteria for both groups suggested performing an ultrasound to confirm the suspicion of VOD in adults and confirm hepatomegaly and ascites in pediatric patients to not only obtain an early diagnosis but also to facilitate a possible differential diagnosis. Since 1997 some ultrasound parameters (seven morphological parameters: hepatomegaly, ascites, thickening of the gallbladder, portal vein diameter, paraumbilical vein patency and splenomegaly and 7 Doppler parameters: portal vein flow, flow direction, portal vein velocity, portal vein flow direction, paraumbilical vein flow, hepatic vein flow demodulation, hepatic artery resistance index) have been described for the prediction, diagnosis and prognostic evaluation of VOD in adult patients (49). However, each single parameter showed poor accuracy, and even when used in a total score (more than 6 criteria), they had sensitivity and specificity never greater than 80%. Recently (50), only 6 of the 14 Lassau parameters showed greater sensitivity and specificity and required less time than analyzing Lassau’s criteria. However, ultrasonography and Doppler ultrasound, even if they have shown good accuracy, may be helpful to confirm/exclude the diagnosis of VOD only when clinical signs of portal hypertension are already present. It should also be noted that, mainly in pediatric patients, the alteration of Doppler parameters may occur later when the typical clinical signs of VOD are present, limiting the usefulness of Doppler ultrasound to achieve an early diagnosis (17).

Regarding the timing of the ultrasound, EBMT suggested (27) performing an ultrasound before HSCT in all patients to have a basis for comparing changes after HCT in suspected VOD. Also, outside of clinical trials, ultrasound monitoring after HSCT, so far, is only performed on request in case of clinical suspicion of VOD. However, in Lassau’s study, where ultrasound was performed weekly after HCT for 1 month, ultrasound parameters changed from baseline (pre-HSCT) only after the first clinical signs of portal hypertension appeared, allowing early confirmation of VOD diagnosis. Recently, in the hepatological field, vibration-controlled transient elastography (FibroScan^®^, Echosens, Paris), a new non-invasive technique, has been developed to evaluate the rigidity of the liver as an expression of liver health (51). In the beginning, FibroScan has been clinically evaluated to assess the degree of hepatic fibrosis. Subsequently, research has shown that changes in liver stiffness have also been observed in other conditions (cholestasis, congestion, inflammation, necrosis, portal hypertension) (52–55). Based on these data, elastography was used to evaluate the presence of portal hypertension in different liver diseases, including VOD (56), whose clinical signs are considered an expression of portal hypertension. Following these observations, recently, some studies have evaluated the change of liver stiffness before and after HCT in the adult and pediatric populations to predict the occurrence of VOD (57–59). These authors observed that hepatic stiffness increased significantly compared to baseline values only in patients who later developed VOD, and mainly, this increase occurred before the onset of clinical symptoms, thus allowing a preclinical diagnosis. From a pathogenetic point of view, it could be hypothesized that this behavior of liver stiffness may be due to the ability of elastography to detect even the slightest changes due to congestion and initial necrosis that occurs in the first phase of portal hypertension before the onset of symptoms and even before any ultrasonographic change. It is important to emphasize that hepatic stiffness should be measured at baseline and weekly after HCT, even without clinical signs, for at least 1 month, as correctly agreed by the scientific council. The heterogeneity of the responses between the panelists and the scientific committee in the timing of imaging after HSCT is mainly due to few studies, mainly on the use of hepatic elastography. The upcoming results of a multicenter study called ELASTOVOD study should further clear uncertainty on this issue. (*ClinicalTrials.gov NCT03426358).*

Item 12 - What is the recommended protocol for monitoring patients receiving defibrotide treatment using imaging techniques such as echo Doppler, abdominal ultrasound, and liver elastography (FibroScan)?

#### Panelists’ view and behavior in VOD clinical practice

Item 12 explores how to monitor with imaging techniques (abdominal ultrasound, Doppler ultrasound and liver elastography) transplant patients. Most of the panelists (65%) answered they based the decision on clinical VOD parameters; 20% performing imaging techniques weekly until day 21 after the start of defibrotide; 6% performing imaging twice a week for 21 days, 3% every 48 hours until the end of treatment, and 6% other. The latter group (other) included the following strategies about imaging use for monitoring: i) once a week until the disease improved, ii) measurement of hepatic venous pressure gradient (HVPG) one week after treatment start and ultrasound monitoring as well, iii) imaging assessment (Ultrasound/Elastography) according to the trend of the biochemical parameters.

#### Comments from the scientific steering committee

The scientific board had no uniform position on the monitoring of defibrotide-treated patients, even if it recognized the need to follow the patients by imaging rather than only clinically.

The duration of defibrotide treatment and its monitoring are still debated. Indeed, in clinical trials, defibrotide was continued until complete response (CR), on average 21.5 days, and CR was defined as the reduction of bilirubin to <2 mg/dl, improvement or resolution of VOD symptoms, or survival to day +100 after HCT (60, 61). However, scarce data exists regarding the relapse rate in case of premature discontinuation due to symptom resolution or how to monitor patients during treatment (33). In recent papers, Doppler ultrasounds performed at baseline and weekly after defibrotide administration have been used successfully to monitor patients and predict responses (10). According to the above intrinsic characteristics, elastography has also been used to monitor the treatment response in recent case reports (17, 52, 62, 63). It has been shown that after treatment, the values of hepatic stiffness decreased in parallel with those of bilirubin in patients who responded to treatment.

Some case reports showed that liver stiffness remained elevated in non-responders, and authors suggest to stop treatment when either ultrasound parameters or liver stiffness return to baseline, and CR is reached, regardless of the duration of treatment. The heterogeneity of the panelists’ responses reflects the need for more solid data on this issue and no definitive answer can be done at the moment on this issue. Therefore, both panelists and the scientific committee look forward to more extensive studies to validate these preliminary observations and identify factors that may predict which patients might have a shorter defibrotide treatment course.

### Area 5 - compliance with product sheet guidelines for managing defibrotide

The questions for Area 5 are focused on adhering to the “summary of product characteristics” for managing Defibrotide’s therapy. In particular, the questions explore how the specialists manage the treatment in the case of an early response within 7 days from the start of the therapy and in the case of SOS/VOD persistence after 21 days of treatment.

Item 13 - In patients exhibiting an early response to defibrotide treatment within 7 days, what is the most appropriate clinical management strategy?

Item 14 - In the event of persistent SOS/VOD after the standard 21 days of treatment with defibrotide, what is the appropriate clinical management strategy?

#### Panelists’ view and behavior in VOD clinical practice

Almost half of the experts participating in the survey stated that in case of early response (within 7 days) from the start of treatment with Defibrotide, they would continue the therapy at full dose up to 21 days, regardless of bilirubin values, according with the “summary of product characteristics”. On the other hand, 38% of the specialists consider suspending the therapy before the 21 days envisaged by the summary of product characteristics, but only in the presence of a few risk factors. Conversely, 9% of the specialists, if the patient achieved an early response, would continue Defibrotide for another 7 days and then stop treatment if the bilirubin levels were normal. Finally, 6% of specialists said they would continue a full dose of Defibrotide until bilirubin levels are normal. In the case of persistent SOS/VOD after the standard 21 days of treatment with Defibrotide, over 50% of panelists declare that they would continue the treatment for up to 10 additional days. Fifteen percent of the specialists would perform a liver biopsy instead. Six percent of the panelists would continue treatment with a halved dosage until the resolution of the SOS/VOD, and 9% would stop the treatment instead. Finally, about a fifth of the specialists preferred to add a personal comment to the 4 proposed response options described. In detail, a couple of them state that they will continue the treatment for up to 10 additional days but also perform other diagnostic investigations, including liver biopsy. Still, others suggest doing a liver biopsy only if feasible based on the bleeding risk. In contrast, one expert suggests performing a trans-femoral liver biopsy if the platelet count is low. Finally, one panelist stated that he would use rtPA-Actilyse in those patients who present with portal flow reversal and/or clinical worsening by suspending Defibrotide, with close monitoring for the bleeding risk.

#### Comments from the scientific steering committee

Several studies have shown the close relationship between the efficacy of Defibrotide treatment and its timely initiation (64). In this regard, it is interesting to note that GITMO recommendations (8) strongly suggest that the treatment start would coincide with the definitive diagnosis of VOD, and they recommend that drug dosage should be used as stated in the Summary of product characteristics (SPC).

Although no specific questions have been asked of the panelist on this issue, the scientific steering committee emphasizes the importance of adherence to the “summary of product characteristics” for managing defibrotide therapy regarding the dose. The investigation involving the Panelist shows a relevant discordance of behavior concerning the indications reported in the “summary of product characteristics”. This discordance regards the fact that 50% of Panelists are in favor of applying in clinical practice an early discontinuation of Defibrotide before the envisaged 21 days, in case of quick normalization of Bilirubin levels, especially in patients with few VOD risk factors. The scientific steering committee emphasizes the importance of adherence to the “summary of product characteristics” for the management of Defibrotide therapy, which provides for a duration of treatment of 21 days, regardless of the achievement of an early response or the bilirubin levels. Indeed, early treatment discontinuation is correct only if the diagnosis of SOS/VOD is not confirmed. Even though some trials are investigating the feasibility of early discontinuation of defibrotide, at present, no data supports the usefulness of defibrotide early discontinuation. Therefore, it should be discouraged, as well as a reduction in the dose of the drug (33).

In the case of persistent SOS/VOD after the standard 21 days of treatment with Defibrotide, the Panelist behavior resulted in extremely heterogeneous, as reported above. The failure of therapy with Defibrotide means a dismal prognosis and absence of effective therapies. The scientific steering committee emphasizes the need to improve the diagnostic workup, especially when there is no response to standard therapy. A proper diagnosis allows the most appropriate and effective treatment for patients. It should always be re-evaluated in the case of SOS/VOD not responsive to the standard treatment with Defibrotide. In this regard, at most 20% of the panelists considered re-evaluation of VOD/SOS diagnosis a priority. Indeed, the report shows that, in clinical practice, many specialists arbitrarily adopt several therapeutic interventions, including rtPA-Actilyse, without performing an appropriate diagnostic re-evaluation of VOD; however, regarding the use of rtPA-Actilyse the major part of panelists were completely disagree.

Item 15 - What concomitant therapies are considered or recommended when administering defibrotide?

#### Panelists’ view and behavior in VOD clinical practice

Ninety-five percent of the survey participants indicated that, in VOD, in addition to defibrotide, other treatments are appropriate. These treatments are not conventional support treatments (such as diuretics and supportive care with red blood cells and platelet concentrates). They are represented by ursodeoxycholic acid, N-acetylcysteine, steroids, and platelet transfusion.

#### Comments from the scientific steering committee

This high percentage of panelists declaring additional unstandardized therapies underlines the physician worries for the poor prognosis of VOD, especially for severe and very severe forms; the Overall Survival remains far below what can ‘be expected in patients not affected by VOD (61% versus 90-95%) (65).

Since other active agents are also available alongside defibrotide, it is reasonable to propose combined systemic treatments based on the synergy between them. However, defibrotide remains the only agent approved by regulatory bodies. The drugs indicated in our survey as possible concomitant treatments in association with defibrotide were Steroids, Acetylcysteine and Ursodeoxycholic acid. Steroids were used by different Authors although with conflicting results on outcome and dosage (high vs low) (14, 66–70). Acetylcysteine (NAC) has been used in different adult and pediatric trial but with inconclusive results (71–73). Similarly, the usefulness of the association of NAC with defibrotide has yet to be studied in a controlled study.

Ursodeoxycholic acid (UDCA) has pleiotropic effects and modulates inflammation and cellular apoptosis. UDCA is routinely recommended in the transplant patient as it can reduce the incidence of jaundice, intestinal and hepatic GVHD, as well as Not Relapse Mortality (21) and therefore, most patients who develop VOD should be already on treatment with UDCA. The beneficial effects of UDCA are evident in transplant patients even at a long-term follow-up (22), and there are no reasons to discontinue the administration of UDCA even after the diagnosis of VOD. It should be noted, however, that other active agents can be associated with defibrotide besides those indicated in our survey. The association of AT-III with defibrotide in the treatment of VOD has shown encouraging results. A combination treatment of defibrotide plus antithrombin III (74) has been implemented in a small number of patients who presented VOD with better results than could have been expected with defibrotide alone. In fact, in this study, all 14 patients with VOD who received combined therapy achieved complete remission, and 93% (13/14) survived until day +100. Another drug that is active and could be combined with defibrotide is thrombomodulin (TM). Thrombomodulin has multiple beneficial actions in diseases in the pathogenesis of which endothelial damage enters it reduces PAI, has an inhibiting effect on F-VIII and F-V activation (PC dependent mechanisms) and actions independent from activated PC (anticomplementary, anti-inflammatory effects). In addition to treating sepsis, thrombomodulin has been demonstrated to be active in experimental models of VOD. It has been used as a treatment for VOD arising after hematopoietic transplantation alone or in association with 6MP or also in association with defibrotide. The largest series described, in a retrospective multicenter study, 41 Japanese patients treated with TM for VOD arising after transplantation (380 IU/kg/day for 8 days) with a response rate comparable to those that can be obtained with defibrotide, CR of 54% and OS of 48% at 100 days (75). Thus, the usefulness of these agents in association with defibrotide awaits further prospective and controlled studies, in which the population studied will be well characterized for their prognostic factors (severity according to EBMT criteria, the interval between the fulfilment of the criteria for diagnosis and onset of therapy).

## Summary of final key recommendations

### Evaluation of the patient prior HSCT

**Figure 1:**
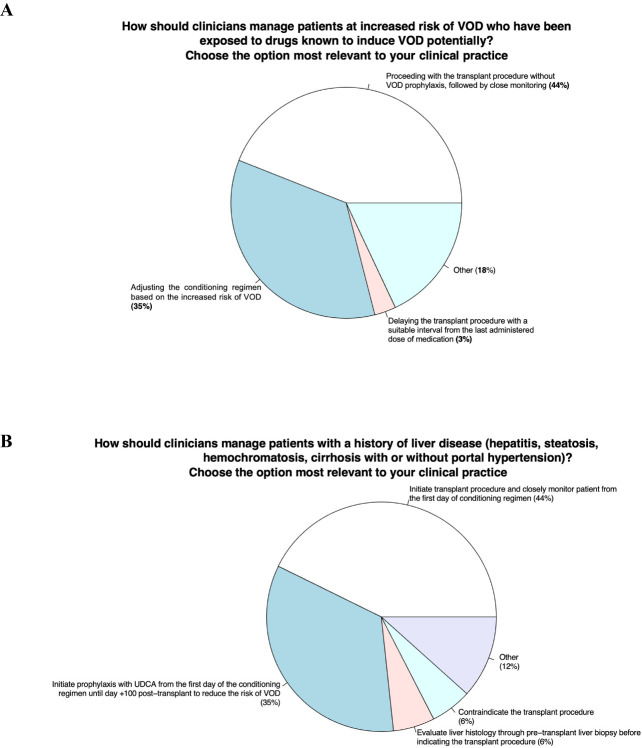
Evaluation of the patient prior HSCT.

1. In patients at increased risk of VOD who have been exposed to drugs known to potentially induce VOD it would be advisable to start the transplantation without prophylaxis but with close follow-up (**Figure 1A**).
2. In patients with a history of liver disease (hepatitis, steatosis, hemochromatosis, cirrhosis with or without portal hypertension) it would be advisable to start the transplantation with a close follow-up (**Figure 1B**).

### Identification of clinical-laboratory aspects for defining a correct diagnosis and subsequent appropriate therapy

**Figure 2:**
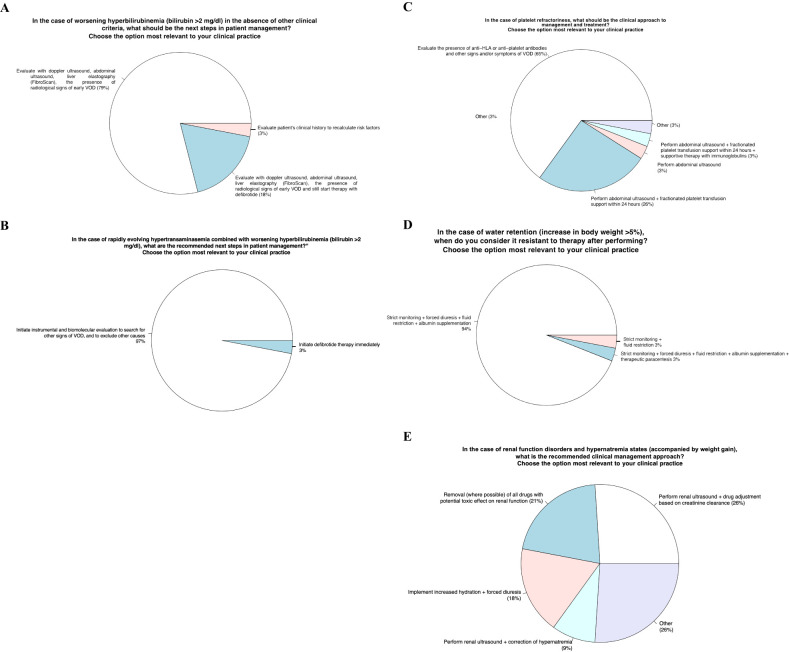
Identification of clinical-laboratory aspects for defining a correct diagnosis and subsequent appropriate therapy.

3. In case of worsening hyperbilirubinemia (bilirubin >2mg/dl) in the absence of other clinical criteria, it would be advisable to evaluate radiological signs of VOD by ultrasound and elastography in the next steps of patient management (**Figure 2A**).
4. In case of rapidly evolving hypertransaminasemia combined with worsening hyperbilirubinemia (bilirubin >2 mg/dl), it would be advisable to carry out further imaging and laboratory investigations to evaluate other signs of VOD and possibly exclude other causes in the next steps of patient management (**Figure 2B**).
5. In case of platelet refractoriness, the clinical approach to management and treatment would be to assess the presence of antibodies anti-HLA or anti-platelets and other VOD signs or symptoms (**Figure 2C**).
6. In case of water retention (increase in body weight >5%), the patients should undergo closing monitoring, high diuretic dose administration, fluid restriction intake and albumin infusion (**Figure 2D**).
7. In case of renal function disorders and hypernatremia states accompanied by weight gain, it would be advisable to perform renal ultrasound and adjustment of the drug according to creatinine clearance, to withdraw nephrotoxic drugs and to increase fluid hydration together to high diuretic dose administration (**Figure 2E**).

### Integration between clinical evaluations and EBMT criteria

**Figure 3:**
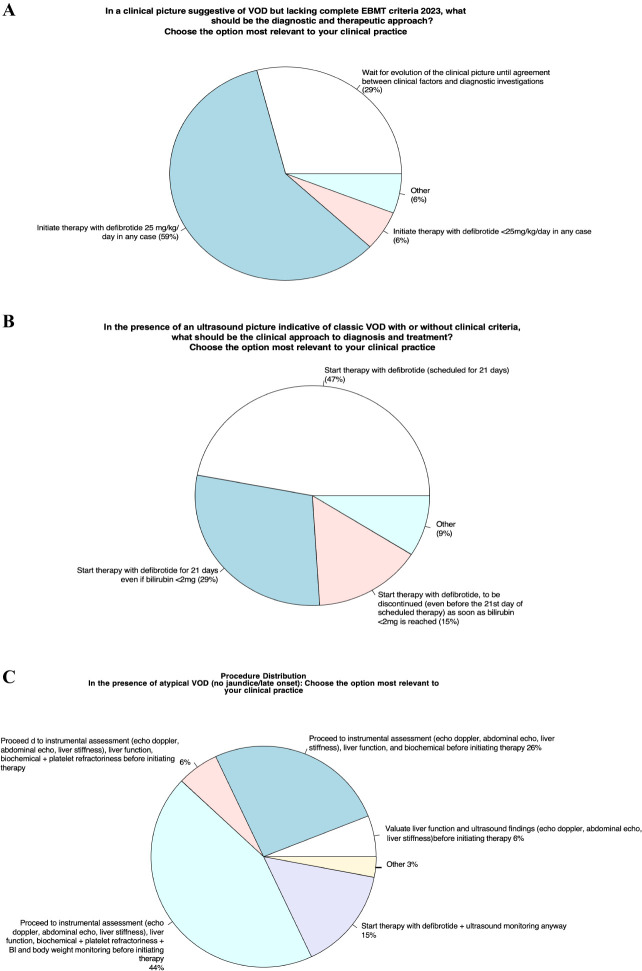
Integration between clinical evaluations and EBMT criteria.

8. In a critical picture suggestive of VOD but lacking complete EBMT criteria 2023, it would be advisable to start defibrotide treatment anyway (**Figure 3A**).
9. In presence of an ultrasound picture indicative of classic VOD with or without clinical criteria, it would be advisable to start treatment with defibrotide (**Figure 3B**).
10. In presence of atypical VOD (no jaundice/late onset) it would be advisable to have further evaluation by imaging (abdominal ultrasound, Doppler ultrasound, and elastography) and laboratory tests (including assessment for the presence of platelet refractoriness) and body weight monitoring to confirm the diagnosis of VOD sooner to start with a specific treatment (**Figure 3C**).

### Role of monitoring with imaging techniques (doppler ultrasound, abdominal ultrasound, liver elastography) in transplant patients and those undergoing defibrotide therapy

**Figure 4:**
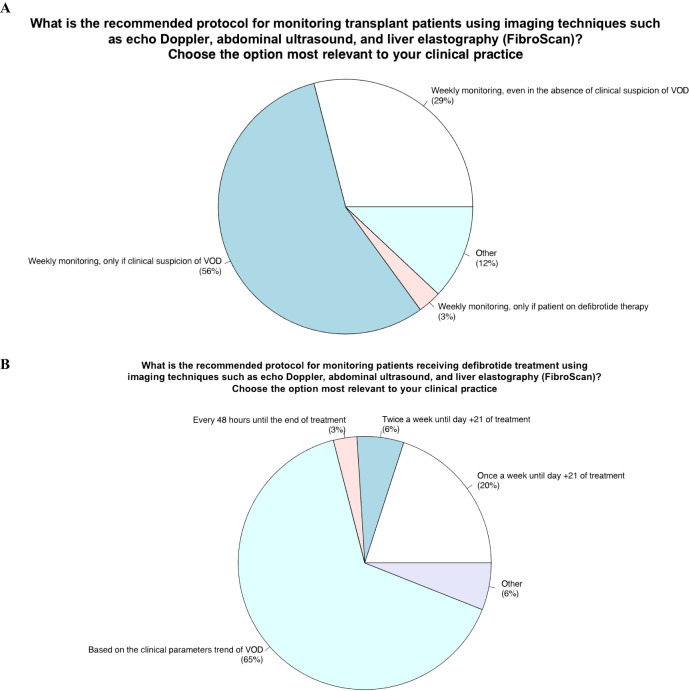
Role of monitoring with imaging techniques (doppler ultrasound, abdominal ultrasound, liver elastography) in transplant patients and those undergoing defibrotide therapy.

11. The recommended protocol for monitoring transplant patients using imaging techniques such as echo Doppler, abdominal ultrasound, and liver elastography (FibroScan) would be to perform once-a-week evaluation, but only if clinical suspicion of VOD is present (**Figure 4A**).
12. The protocol for monitoring patients receiving defibrotide treatment using imaging techniques such as echo Doppler, abdominal ultrasound, and liver elastography FibroScan) should be based on clinical VOD parameters (**Figure 4B**).

### Compliance with product sheet guidelines for managing defibrotide

**Figure 5:**
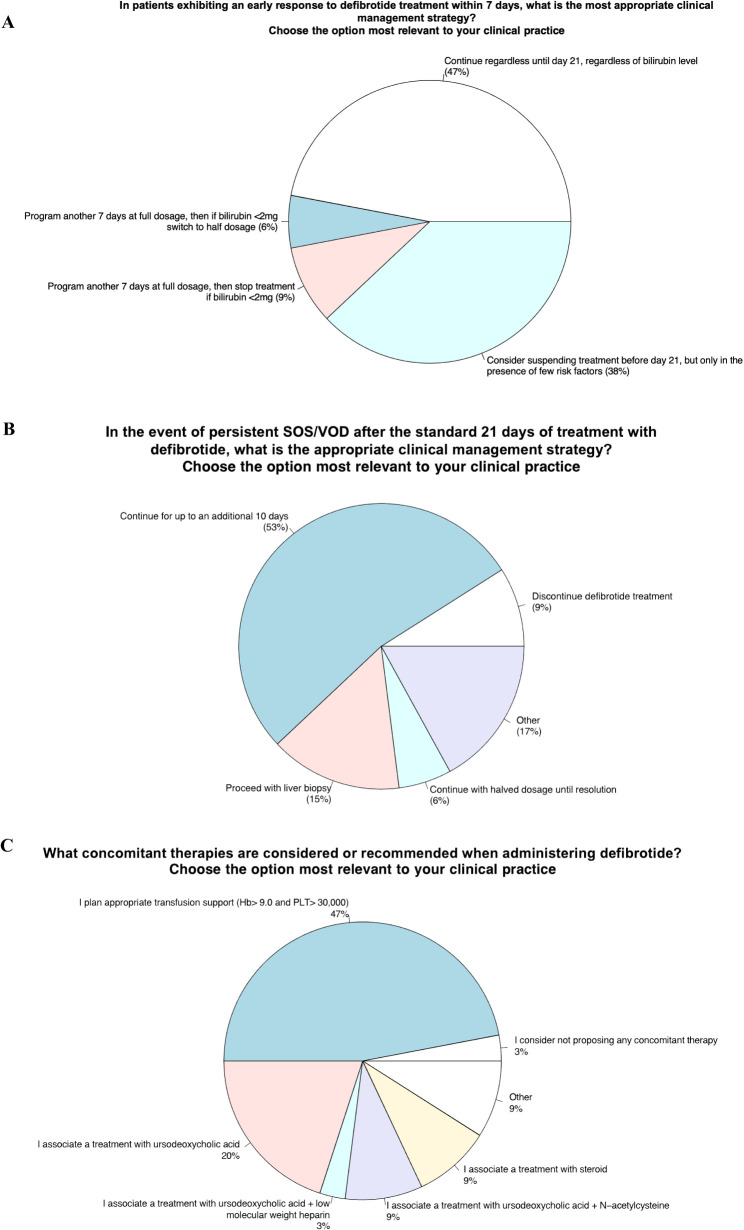
Compliance with product sheet guidelines for managing defibrotide.

13. In patients exhibiting an early response to defibrotide treatment within 7 days, it would be advisable to continue the therapy at full dose up to 21 days, regardless of bilirubin values, according with the “summary of product characteristics”. Alternatively, it would also suggested suspending the therapy before the 21 days envisaged by the summary of product characteristics, but only in the presence of a few risk factors (**Figure 5A**).
14. In case of persistent SOS/VOD after the standard 21 days of treatment with defibrotide, it would be advisable to continue the treatment for up to 10 additional days (**Figure 5B**).
15. In VOD, in addition to defibrotide, other treatments, such as ursodeoxycholic acid, N-acetylcysteine, steroids, and platelet transfusion would be considered (**Figure 5C**).

## Conclusion

The purpose of this Delphi project stems precisely from an awareness of the diagnostic and therapeutic difficulties associated with VOD, a complex and adaptable disease in the transplant setting. The analysis of the proposed items in this study reveals the discordance among panelists, highlighting the challenges faced despite the presence of guidelines and position papers. A multidisciplinary approach is mandatory to address these difficulties, emphasizing the collaboration between hematologists, hepatologists, and radiologists. This collaboration is crucial for standardizing reports, improving the caliber of technologies, and exchanging knowledge. The project highlights the relevance of multidisciplinary diagnosis in the decision-making process of VOD, which will only grow in significance with evidence-based therapy approach. It is important to note that, thanks in part to the implementation of the multicenter Italian ELASTOVOD study, many transplant centers in Italy are now equipped with cutting-edge imaging technologies and have experienced and dedicated consultants. This study has played a significant role in spreading the adoption of these advanced imaging technologies throughout the country. However, this study demonstrates that the diagnostic methods for VOD need to be more consistent throughout the Italian territory. Therefore, establishing a network spearheaded by the Italian Group for Bone Marrow Transplantation (GITMO) and the Italian Association of Pediatric Hematology Oncology (AIEOP) is paramount. This comprehensive network will assist clinical centers needing more technical expertise and ensure sufficient control over VOD.

## Data Availability

The original contributions presented in the study are included in the article/supplementary material. Further inquiries can be directed to the corresponding author.

## DELPHI-VOD expert panel

Edoardo Benedetti, UO Ematologia, Pisa (PI); Fabio Benedetti, CTMO, Verona (VR); Paolo Bernasconi, Fondazione IRCCS Policlinico San Matteo UOC Ematologia, Pavia (PV); Alessandro Busca, SSD Trapianto cellule staminali, Torino (TO); Elisabetta Calore, Azienda Ospedaliera-Università di Padova, Padova (PD); Maurizio Caniglia, SC di Oncoematologia Pediatrica, Perugia (PG); Michele Carella, IRCCS CASA SOLLIEVO DELLA SOFFERENZA, San Giovanni Rotondo (FG); Michele Cimminiello, A.O.R. San Carlo di Potenza, UOC di Ematologia con TMO, Potenza (PZ); Nicola Di Renzo, PO “Vito Fazzi” - ASL LECCE, Lecce (LE); Luca Facchin, Arcispedale Santa Maria Nuova, Reggio nell’Emilia (RE); Anna Grassi, Centro trapianto di midollo osseo, Bergamo (BG); Massimo Martino, Centro Trapianti Midollo Osseo e Terapie Cellulari, GOM BMM, Reggio di Calabria (RC); Patrizio Mazza, EMATOLOGIA, Taranto (TA); Maria Crsitina Menconi, Oncoematologia Pediatrica, Pisa (PI); Nicola Mordini, Sc ematologia AO S Croce e Carle, Cuneo (CN); Pellegrino Musto, UOC Ematologia con Trapianto, AOUC Policlinico, Bari (BA); Francesco Onida, Fondazione IRCCS Ca’ Granda Ospedale Maggiore Policlinico, Università degli Studi di Milano, Milano (MI); Fabrizio Pane, Università Federico II di Napoli, Napoli (NA); Domenico Pastore, Ematologia, Brindisi (BR); Francesca Patriarca, Clinica Ematologica e Centro Trapianti ASUFC, Udine (UD); Katia Perruccio, SC di Oncoematologia Pediatrica, Perugia (PG); Antonio Piroddi, CTMO Ospedale Microcitemico, Cagliari (CA); Lucia Prezioso, EMATOLOGIA E CTMO, Parma (PR); Anna Proia, AO San Camillo, Roma (RM); Marco Rabusin, IRCCS Burlo Garofalo, Trieste (TS); Riccardo Saccardi, SODc Terapie cellulari e Medicina Trasfusionale, Firenze (FI); Nicole Santoro, UOC Ematologia Clinica, Dipartimento di Oncologia e ematologia, Ospedale Civile di Pescara, Pescara (PE); Barbara Sarina, istituto clinico humanitas, Rozzano (MI); Carmine Selleri, AOU San Giovanni di Dio e Ruggi d’Aragona, Salerno (SA); Cristina Skert, Ospedale dell’Angelo, Venezia (VE); Paolo Tambaro, AORN Santobono Pausilipon, Napoli (NA); Veronica Tintori, AOU Meyer, Firenze (FI); Giuseppe Visani, Ematologia &Centro Trapianti Pesaro, Pesaro (PU); Francesco Zalio, Azienda Ospedaliera SS Antonio e Biagio, Alessandria (AL).
